# Vitamin D supplementation of initially vitamin D‐deficient mice diminishes lung inflammation with limited effects on pulmonary epithelial integrity

**DOI:** 10.14814/phy2.13371

**Published:** 2017-08-03

**Authors:** Shelley Gorman, Alysia G. Buckley, Kak‐Ming Ling, Luke J. Berry, Vanessa S. Fear, Stephen M. Stick, Alexander N. Larcombe, Anthony Kicic, Prue H. Hart

**Affiliations:** ^1^ Telethon Kids Institute University of Western Australia Subiaco Western Australia Australia; ^2^ Centre of Microscopy Characterisation and Analysis The University of Western Australia Nedlands Western Australia Australia; ^3^ Department of Respiratory Medicine Princess Margaret Hospital for Children Perth Western Australia Australia; ^4^ School of Paediatrics and Child Health The University of Western Australia Nedlands Western Australia Australia; ^5^ Centre for Cell Therapy and Regenerative Medicine School of Medicine and Pharmacology The University of Western Australia and Harry Perkins Institute of Medical Research Nedlands Western Australia Australia; ^6^ Occupation and Environment School of Public Health Curtin University Perth Western Australia Australia

**Keywords:** Epithelium, inflammation, integrity, lung, mice, vitamin D

## Abstract

In disease settings, vitamin D may be important for maintaining optimal lung epithelial integrity and suppressing inflammation, but less is known of its effects prior to disease onset. Female BALB/c dams were fed a vitamin D_3_‐supplemented (2280 IU/kg, VitD^+^) or nonsupplemented (0 IU/kg, VitD^−^) diet from 3 weeks of age, and mated at 8 weeks of age. Male offspring were fed the same diet as their mother. Some offspring initially fed the VitD^−^ diet were switched to a VitD^+^ diet from 8 weeks of age (VitD^−/+^). At 12 weeks of age, signs of low‐level inflammation were observed in the bronchoalveolar lavage fluid (BALF) of VitD^−^ mice (more macrophages and neutrophils), which were suppressed by subsequent supplementation with vitamin D_3_. There was no difference in the level of expression of the tight junction proteins occludin or claudin‐1 in lung epithelial cells of VitD^+^ mice compared to VitD^−^ mice; however, claudin‐1 levels were reduced when initially vitamin D‐deficient mice were fed the vitamin D_3_‐containing diet (VitD^−/+^). Reduced total IgM levels were detected in BALF and serum of VitD^−/+^ mice compared to VitD^+^ mice. Lung mRNA levels of the vitamin D receptor (VDR) were greatest in VitD^−/+^ mice. Total IgG levels in BALF were greater in mice fed the vitamin D_3_‐containing diet, which may be explained by increased activation of B cells in airway‐draining lymph nodes. These findings suggest that supplementation of initially vitamin D‐deficient mice with vitamin D_3_ suppresses signs of lung inflammation but has limited effects on the epithelial integrity of the lungs.

## Introduction

The airway epithelium is a defensive barrier against potential irritants, allergens, and pathogens that may cause lung dysfunction. The epithelial barrier consists of epithelial cells held together by tight junction proteins (zona occludens, claudin, adherens), adhesion junction proteins (E‐cadherin), junction adhesion molecules, gap junctions, and connexions that work in unison to maintain the protective barrier (Georas and Rezaee 2014). Disruption of the airway epithelium is thought to contribute toward the pathogenesis of chronic lung diseases including asthma (de Boer et al. 2008; Xiao et al. 2011; Hackett et al. 2013), chronic obstructive pulmonary disease (Shaykhiev et al. 2011; Heijink et al. 2012), and cystic fibrosis (Godfrey et al. 1993; Coyne et al. 2002; Castellani et al. 2012). Vitamin D is an essential hormone for bone health, and is under consideration as a modulator of other biological processes, including epithelial function and immunity. The potential for vitamin D to alter epithelial function has been highlighted in a number of mechanistic studies (and reviewed below).

Vitamin D is usually obtained through skin exposure to ultraviolet B radiation present in sunlight, whereby the skin precursor 7‐dehydrocholesterol is converted into vitamin D_3_. It is further converted into 25‐hydroxyvitamin D (25(OH)D) and then 1,25‐dihydroxyvitamin D (1,25(OH)_2_D) through a series of hydroxylation events in the liver and kidneys (respectively). 1,25(OH)_2_D is the most “active” form of vitamin D, and classically acts through the genomic vitamin D receptor (VDR), and nonclassically through a surface receptor (reviewed in Hii and Ferrante 2016). Vitamin D is also obtained through dietary sources and supplements. Animal modeling studies have demonstrated the potential of dietary vitamin D or the VDR to regulate the integrity of epithelium of the lungs (Li et al. 2015; Zhang et al. 2015a; Fischer et al. 2016; Shi et al. 2016), gut (Kong et al. 2008; Zhao et al. 2012; Liu et al. 2013; Ooi et al. 2013; Assa et al. 2014; Du et al. 2015; Raftery et al. 2015; Kuhne et al. 2016; Villa et al. 2016), skin (Bikle et al. 2004; Oda et al. 2009; Hong et al. 2010), eyes (Elizondo et al. 2014), and liver (Firrincieli et al. 2013).

Active 1,25(OH)_2_D and expression of the VDR can improve airway barrier function, and reduce lung disease and injury in mice (Liu et al. 2013; Lam et al. 2015). Treatment of BALB/c mice with active 1,25(OH)_2_D (100 ng intraperitoneal injection) altered the expression of zona occludens‐1 (ZO‐1) and E‐cadherin in airway epithelial cells, and reduced signs of asthma induced by dermal sensitization and respiratory challenge with toluene di‐isocyanate (Li et al. 2015). E‐cadherin expression was also increased in the lung epithelial cells of mice supplemented with high levels of vitamin D (10,000 IU/kg diet), who exhibited reduced signs of asthma induced by sensitization and challenge with a mixture of clinically relevant antigens, compared to mice fed a vitamin D‐sufficient diet (2000 IU/kg diet) (Fischer et al. 2016). 1,25(OH)_2_D also improved the integrity of bronchial epithelial cells in vitro (Li et al. 2015; Zhang et al. 2015a). VDR^−/−^ mice had reduced ZO‐1 and occludin protein levels in the lungs, as well as increased lung epithelial permeability, in comparison to wild‐type mice following LPS challenge (Shi et al. 2016). Treatment of VDR^−/−^ mice with an analog of 1,25(OH)_2_D, paricalcitol, reduced lung injury and epithelial integrity defects induced by LPS, suggesting that there may be redundancy in the ability of 1,25(OH)_2_D to regulate lung epithelial integrity (independently of the VDR) (Shi et al. 2016).

Vitamin D may modulate epithelial function through its capacity to suppress the development of inflammation. Reduced peribronchial inflammation and improved epithelial function were observed in mice with toluene di‐isocyanate‐induced asthma treated with 1,25(OH)_2_D (Li et al. 2015). There were also suppressive effects of vitamin D on proinflammatory cytokine expression and inflammatory cell infiltration into the lungs of mice with allergic airway disease (Fischer et al. 2016) or injury (Shi et al. 2016), which were associated with improved epithelial integrity. Increased lung inflammation also occurred in VDR^−/−^ mice with lung injury compared to wild‐type mice (Shi et al. 2016).

While there is emerging evidence that vitamin D is essential for maintaining epithelial integrity and impairing the development of inflammation in the lungs during disease (Li et al. 2015; Fischer et al. 2016; Shi et al. 2016), less is known about its effects prior to disease onset. In the current study, we explored the potential for dietary vitamin D to preserve epithelial integrity and limit inflammation in the lungs prior to the onset of disease. We have recently reported that serum levels of 25(OH)D inversely correlated with total bacterial and *Pseudomonas* operational taxonomic units (OTUs) in the lungs of naïve BALB/c mice (Roggenbuck et al. 2016). More neutrophils and macrophages were identified in the BALF of vitamin D‐deficient male mice, which could have been caused by the increased bacterial burden detected in these mice (Roggenbuck et al. 2016). Dietary vitamin D also reduced airway inflammation, but did not compromise lung function in male BALB/c mice with allergic airway disease (using ovalbumin as the experimental allergen) (Gorman et al. 2012b, 2013). In the current study, we hypothesized that dietary vitamin D may maintain epithelial integrity to prevent airway inflammation. Here, we examined the effects of vitamin D deficiency, and dietary supplementation of initially deficient mice, on epithelial integrity in the lungs of otherwise naïve male BALB/c mice.

## Methods

### Mice and diets

**Figure 1:**
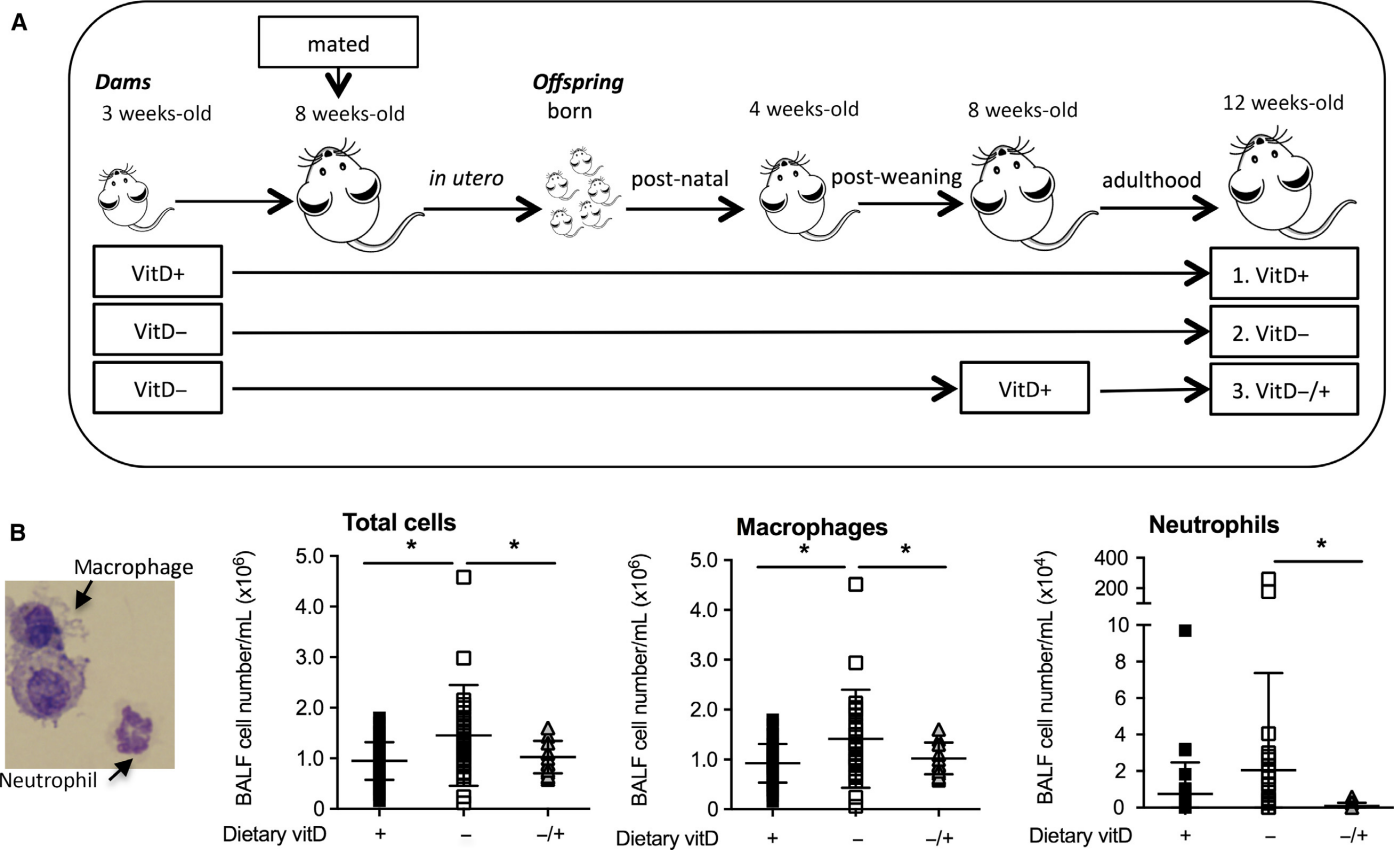
The dietary intake of mice in each treatment, and their effect on major bronchoalveolar lavage fluid cell subtypes. In (A), female BALB/c mice (dams) were fed vitamin D‐supplemented (VitD^+^) or vitamin D‐nonsupplemented (VitD^−^) diets from 3 weeks of age and used to produce offspring. A subgroup of vitamin D‐deficient offspring were fed a vitamin D‐supplemented diet from 8 weeks of age (VitD^−/+^). In (B), total, macrophage and neutrophil cell numbers in bronchoalveolar lavage fluid (BALF) from these offspring at 12 weeks of age are shown with macrophages and neutrophils identified in a representative cytospin of BALF (×100 magnification). Data are shown as mean ± SD (*P < 0.05; two‐way *t* test) for ≥18 mice per treatment. The results for macrophage and neutrophil numbers were partially reported in a previous publication (Roggenbuck et al. 2016) with an additional 3–5 mice per treatment added from a new experiment.

Experiments were performed according to the ethical guidelines of the National Health and Medical Research Council of Australia and with approval from the Telethon Kids Institute Animal Ethics Committee (AEC#238). Mice were purchased from the Animal Resources Centre, Western Australia and maintained under specific pathogen‐free conditions. Experiments were completed in animals between October 2012 and December 2013. All mice were caged in open‐topped cages under 12 h:12 h standard light:dark conditions at 23.0 ± 1.0°C (mean ± SD). Mice were euthanized by anesthetic overload with isoflurane, with blood collected immediately posteuthanasia via cardiac puncture. The fluorescent lighting in animal holding rooms did not emit any detectable ultraviolet B radiation (as measured using an UVX Digital Radiometer, Ultraviolet Products Inc., Upland, USA radiometer), with Perspex plastic covers used as an additional means to block ultraviolet radiation. Female 3‐week‐old BALB/c mice were randomly fed one of two semipure diets. These were either supplemented with vitamin D_3_ (2280 IU vitamin D_3_/kg, SF05‐34, Specialty Feeds, Perth, Western Australia) or not (0 IU vitamin D_3_/kg, SF05‐033, Specialty Feeds) as described previously (Gorman et al. 2012b, 2013). From 8 weeks of age, female mice were mated with male BALB/c mice. These male mice were fed standard mouse chow until breeding (Specialty Feeds; containing 2000 IU vitamin D_3_/kg). Litter sizes and sex ratios of offspring were similar for those offspring born to dams fed either diet (mean of five pups per litter, equal sex ratio). Male offspring were then fed the vitamin D‐replete (VitD^+^, *n* = 26) or ‐deficient (VitD^−^, *n* = 26) diets for the rest of the experiment, except for some initially vitamin D‐deficient mice that were randomly selected to be switched to a vitamin D‐replete diet from 8 weeks of age (VitD^−/+^, *n* = 20, Fig. 1A). End of experiment body weights were 25.6 ± 2.4 g (from 11 litters, mean ± SD), 25.4 ± 2.8 g (from 14 litters), and 24.7 ± 2.4 g (from 10 litters) for male offspring of the VitD^+^, VitD^−^, and VitD^−/+^ treatments, respectively. We have previously reported that male offspring born to vitamin D‐replete and ‐deficient BALB/c dams have serum 25(OH)D levels ≥50 and <20 nmol/L, respectively (Gorman et al. 2012, 2013b, 2016; Roggenbuck et al. 2016). Similarly, serum 25(OH)D levels rise to ≥50 nmol/L in initially vitamin D‐deficient adult offspring supplemented for 4 weeks with vitamin D_3_ (Gorman et al. 2013, 2016; Roggenbuck et al. 2016) as done for the third treatment group (VitD^−/+^; Fig. 1A). Samples were obtained from 12‐week‐old mice.

### Bronchoalveolar lavage fluid for assessment of inflammatory cells

As previously reported (McGlade et al. 2007), BALF was collected following cardiac perfusion with 5 mL PBS, with a total of 1.2 mL PBS (with 0.2% BSA) flushed into the lungs through the trachea. Three lots of 400 *μ*L (totaling 1.2 mL) were instilled into the lungs to avoid overinflation, with a total of 1 mL recollected. Lavage samples were centrifuged (450*g*, 4°C, 10 min) and the supernatant was stored at −20°C until required for analysis. The cell pellet was counted, and cells (5 × 10^5^ in 100 *μ*L) were then centrifuged onto glass slides and stained using the DIFF‐Quik Stain Set 64851 (Lab Aids, Narrabeen, NSW, Australia) as per the manufacturer's instructions. Differential counts of inflammatory cells were performed whereby at least 300 cells were counted for each sample from ≥3 independent fields of view (×100) in a blinded fashion.

### Measuring the wet and dry weights of lungs

Following perfusion, lungs (without trachea) were removed into 1.5 mL polypropylene tubes and their “wet” weights recorded using a fine electronic balance (Ohaus Analytical Standard, >0.0001 g sensitivity). Lungs were dried overnight in a dehydrator (Sunbeam DT5600 Healthy Food Dryer), and reweighed to determine their “dry” weights.

### Quantification of tight junction protein expression in lungs through immunofluorescence

Under 10 cm H_2_O pressure, 10% buffered formalin (in phosphate‐buffered saline) was instilled through the trachea into the lungs of euthanized mice for 2 h. Lungs were removed and fixed for a further 4 days with 10% buffered formalin. Tissue sections were stained for claudin‐1 or occludin and DAPI using methods previously described (Kicic et al. 2006). Briefly, cells were incubated in a solution of 0.1% Sudan Black B in 70% ethanol to block autofluorescence. Cells were then washed in Tris‐buffered saline (TBS) and antigen retrieval performed by incubation at 90°C for 40 min with citrate buffer (10 mmol/L). Cells were cooled on ice, then washed with TBS. Samples were blocked in 1% BSA (w/v), 10% FBS (v/v), 10% normal goat serum (v/v), 0.1% Triton X‐100 (v/v) prior to incubation with rabbit anti‐mouse claudin‐1 or occludin antibody (2.5 *μ*g/mL; Thermo Fisher Scientific, MA). Cells were washed again with TBS and incubated with anti‐rabbit mAb conjugated to AlexaFluor 488 (10 *μ*g/mL; Thermo Fisher Scientific). Finally, nucleic acids were stained using Hoechst 33342 (Thermo Fisher Scientific) and fluorescent signals visualized using Nikon A1Si confocal microscope (Nikon Plan Apo VC 60× 1.4NA oil immersion objective). ImageJ64 (v1.45s, 2013) was used to quantify expression levels of protein as described previously (Burgess et al. 2010). Briefly, the integrated density of fluorescence was determined for 13 epithelial cells by using the circle tool for a given area for each sample. This score was averaged with the integrated fluorescence density subtracted from the background (mean of three areas) for each sample.

### Measurement of total protein and Ig levels in BALF and serum

Total protein levels were measured in BALF using the Bradford dye‐binding method (Bradford 1976) as described by the manufacturer (Bio‐Rad, Gladesville, NSW, Australia). Immunoglobulin (Ig) levels were measured using antibody pairs and supplied by Southern Biotechnology (AL) for IgA, IgG, and IgM. BALF and serum samples were serially diluted twofold (for serum IgM), respectively, and total IgA, IgG, and IgM were measured using time‐resolved fluorescence as described previously (Scott et al. 2011). Immunoglobulin levels in BALF were nomalized by dividing titers by the concentration of total protein levels in BALF.

### Detection of *Cldn‐1*,* Ocln*, and *Vdr* mRNA in lung cells

Messenger RNA was extracted from 2 mm^3^ finely minced portions of the whole left lung, with cDNA synthesized and real‐time assays performed as described previously using Quantitect Primer Assays (Qiagen, Hilden, Germany) for detection of mRNAs of the claudin‐1 (*Cldn1*), occludin (*Ocln*), and *Vdr* genes with *Eef1α* used as the house‐keeping control as described previously (Gorman et al. 2012a).

### FACS analysis of immune cells

Airway‐draining lymph node cells (posterior mediastinal, tracheobronchial, parathymic, ADLN) were removed from mice and physically disaggregated. Staining of surface antigens was performed as described previously (Gorman et al. 2007) using anti‐rat monoclonal antibodies supplied by BD Biosciences (CD86‐BIO, streptavidin‐PE‐Cy5, B220‐PE‐Cy7, MHC class II‐FITC). At least 10,000 cells of interest were collected using the FACS LSRII (BD Biosciences) flow cytometer. Data were analyzed using FlowJo software (v9.5.2, TreeStar Inc., Ashland OR).

### Statistical analyses

Data were compared using one‐way ANOVA (with Tukey's multiple comparisons post hoc analysis) or an unpaired two‐way Student's *t* test (with Welch's correction for unequal variances if necessary) using the Prism 5 for Mac OS X statistical analysis program. A nonparametric Spearman's test was used to determine the correlation between IgM levels in BALF and serum. Differences were considered significant with a *P *< 0.05.

## Results

### Vitamin D deficiency increased macrophage numbers in BALF of male mice

Increased cell numbers in BALF of naïve male adult BALB/c mice with vitamin D deficiency maintained throughout life (from conception onwards, VitD^−^) were observed, compared to mice fed a vitamin D‐containing diet (VitD^+^, Fig. 1B, *P* = 0.009), when the results of a new experiment (of 3–5 mice/treatment) were combined with our previous published findings (*n* ≥ 18 mice/treatment) (Roggenbuck et al. 2016). This effect was mainly driven by macrophages in the BALF (Fig. 1B, *P* = 0.01). There was also a nonsignificant increase in the numbers of neutrophils (*P* = 0.2) in the BALF of VitD^−^ mice (Fig. 1B) compared to VitD^+^ mice. These effects on macrophage and neutrophil numbers were significantly reversed by subsequent supplementation of initially deficient adult mice for 4 weeks with a diet containing vitamin D_3_ (VitD^−/+^, *P* ≤ 0.04). No lymphocytes or eosinophils were observed in BALF cytospins. These findings suggest low‐level inflammation (e.g., increased macrophages and neutrophils in BALF) occurs in the lungs of vitamin D‐deficient and otherwise naïve mice, which can be suppressed by dietary supplementation with vitamin D.

### Vitamin D deficiency did not affect lung weight

**Figure 2:**
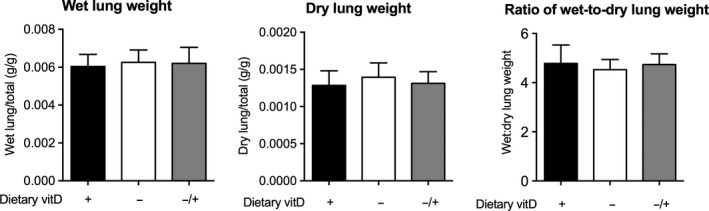
Vitamin D deficiency did not affect the wet or dry lung weights. Female BALB/c mice (dams) were fed vitamin D‐supplemented (+) or vitamin D‐nonsupplemented (−) diets from 3 weeks of age and used to produce offspring. A subgroup of vitamin D‐deficient offspring was fed a vitamin D‐supplemented diet from 8 weeks of age (^−/+^). The wet and dry lung weights relative to total body weight, as well as the ratio of wet‐to‐dry lung weights are shown for all mice at 12 weeks of age (*n* = 15–17 mice/treatment). Data are shown as mean + SD (*P* > 0.05 for all comparisons, one‐way ANOVA).

One means through which vitamin D deficiency could promote the egress of inflammatory cells into the BALF is by affecting the integrity of lung epithelium. To examine whether vitamin D deficiency increased extravascular fluid accumulation in the lungs (pulmonary edema), we compared the wet and dry lung weights, where any deviations in the ratio of the wet‐to‐dry lung weight may represent pulmonary edema (either alveolar and/or interstitial) or tissue damage (Matsuyama et al. 2008). There was no significant effect of vitamin D deficiency or further supplementation with vitamin D_3_ upon the wet or dry lung weight (relative to total body weight) or the wet‐to‐dry lung weight ratio (Fig. 2). These results suggest that there was no significant extravascular fluid leakage in vitamin D‐deficient mice.

### Vitamin D supplementation of initially deficient mice reduced the expression of claudin‐1

**Figure 3:**
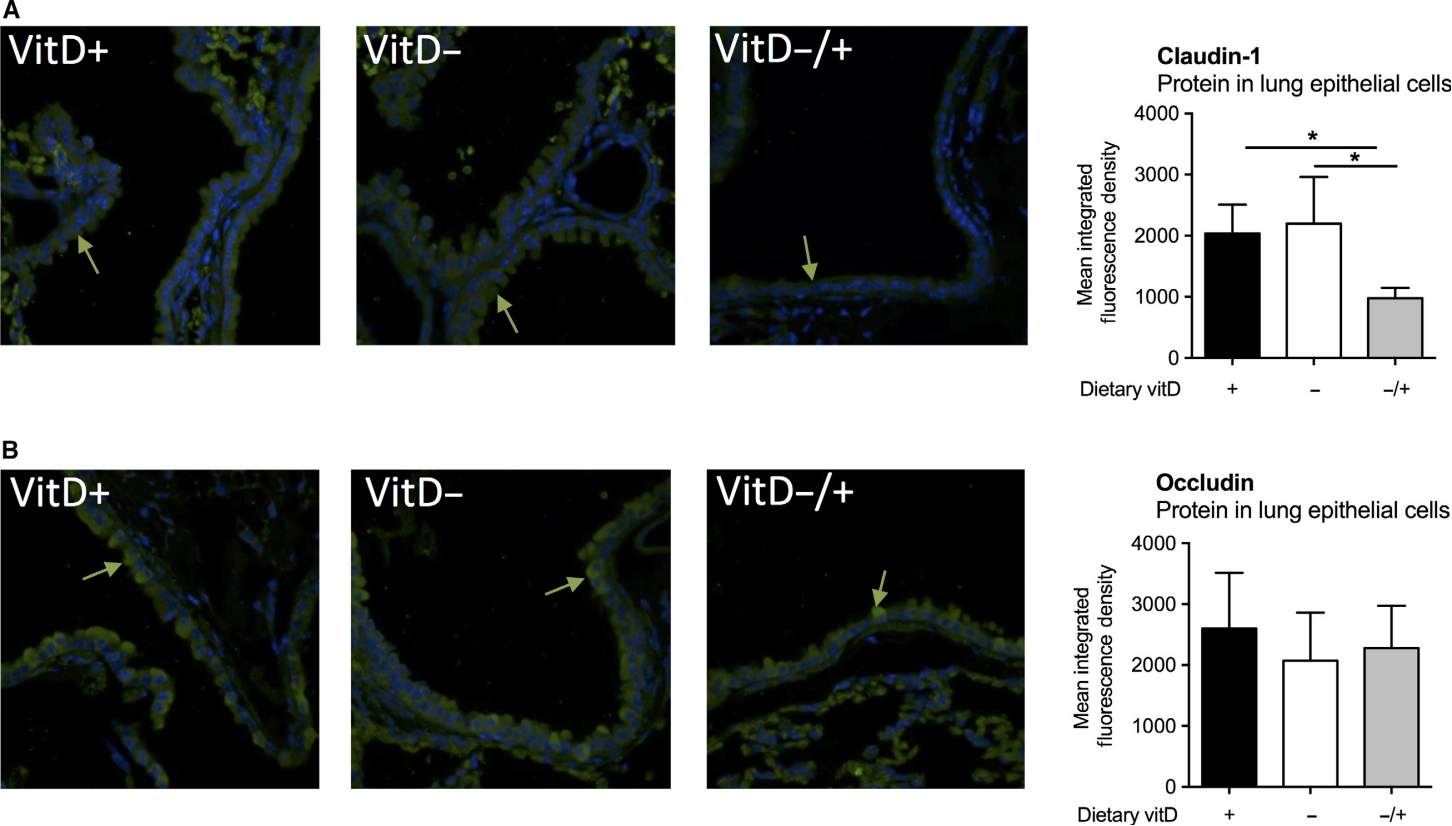
Vitamin D supplementation of initially deficient mice reduced the expression the claudin‐1 but not occludin on lung epithelium. Female BALB/c mice (dams) were fed vitamin D‐supplemented (VitD^+^) or vitamin D‐nonsupplemented (VitD^−^) diets from 3 weeks of age and used to produce offspring. A subgroup of vitamin D‐deficient offspring was fed a vitamin D‐supplemented diet from 8 weeks of age (VitD^−/+^). Representative sections of lungs obtained from 12‐week‐old mice from each treatment were stained with antibodies specific for (A) claudin‐1 and (B) occludin (green arrows indicate binding of green fluorescently tagged antibodies), with DAPI used to identify the nucleus of cells (blue nuclei). Green fluorescence levels were quantified in 13 epithelial cells for three sections (×60 magnification) per sample with the mean for each sample shown (*n* = 5 mice/treatment). Data are shown as mean + SD (*P < 0.05, one‐way ANOVA).

We then examined the expression of the tight junction proteins claudin‐1 and occludin via immunohistochemistry. Claudin‐1 and occludin form the main components of the tight junction, and their expression regulates the diffusion of small molecules between epithelial cells needed for normal barrier function. We did not detect any differences in mRNA levels of *Cldn*‐*1* or *Ocln* in the lungs of mice from any treatment (data not shown). Vitamin D deficiency did not significantly modify the expression of either protein on lung epithelial cells (Fig. 3). However, further dietary supplementation of initially deficient mice with vitamin D_3_ inhibited the expression of claudin‐1 (Fig. 3A: *P* = 0.008 for VitD^−^ compared to VitD^−/+^; *P* = 0.02 for VitD^+^ compared to VitD^−/+^) but not occludin (Fig. 3B, *P* > 0.05) protein as measured by immunohistochemistry.

### Vitamin D deficiency inhibited IgG and IgM levels in BALF, while IgM levels in BALF were not improved by supplementing initially deficient mice with vitamin D

**Figure 4:**
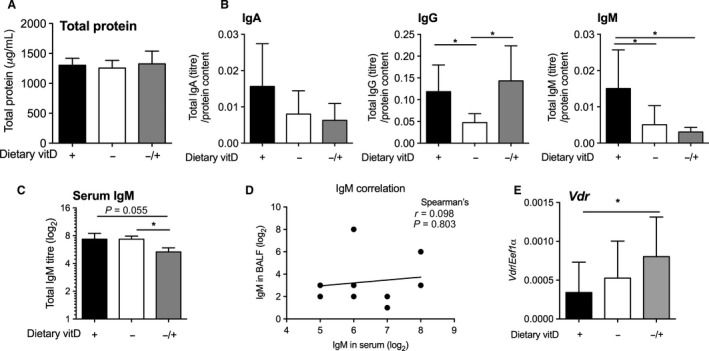
Total protein and antibody isotypes (IgA, IgG, and IgM) in bronchoalveolar lavage fluid, serum IgM levels, and Vdr mRNA in the lungs. Female BALB/c mice (dams) were fed vitamin D‐supplemented (+) or vitamin D‐nonsupplemented (−) diets from 3 weeks of age and used to produce offspring. A subgroup of vitamin D‐deficient offspring was fed a vitamin D‐supplemented diet from 8 weeks of age (^−/+^). In (A), total protein levels (12–17 mice per treatment), and in (B), total IgA, IgG, and IgM levels (9–10 mice per treatment; *P < 0.05, two‐way *t* test) in the BALF (relative to BALF protein levels) of these mice at 12 weeks of age. In (C), total IgM levels in the sera of mice at 12 weeks of age (three mice per treatment; *P < 0.05; two‐way *t* test). In (D), correlation of IgM levels in BALF and serum (Spearman's: *n* = 9). In (E), mRNA levels of the vitamin D receptor (Vdr) in the lungs are shown relative to the house‐keeping gene Eef1α (8‐10 mice per treatment; *P < 0.05; two‐way *t* test). Data are shown as mean + SD.

An alternate means of measuring the integrity of the lung epithelia is via measuring the concentration of protein present in BALF. There was no difference in total protein levels in BALF in any treatment group (Fig. 4A). In the brain, immunoglobulin levels are sometimes measured to assess the degree of nonspecific blood‐to‐brain leakage and capillary permeability (Lam et al. 2015). We examined protein levels of a number of immunoglobulins, specifically IgA, IgG, and IgM, and found that IgM (but not IgA) levels were reduced in VitD^−^ mice and VitD^−/+^ mice compared to VitD^+^ mice (Fig. 4B: IgA – *P* = 0.03 for VitD^+^ compared to VitD^−^, 66% reduction; IgM – *P* = 0.01 for VitD^+^ compared to VitD^−/+^, 80% reduction). Serum levels of IgM were reduced in VitD^−/+^ mice relative to the VitD^+^ (*P* = 0.055) and VitD^−^ (*P* = 0.01) treatments (Fig. 4C). However, we did not observe a significant correlation between serum and BALF IgM levels (Fig. 4D: *P* = 0.803, *r* = 0.098: Spearman's). We also observed reduced total IgG levels (60% reduction) in the BALF of VitD^−^ mice compared to VitD^+^ mice (*P* = 0.005), with this effect reversed by vitamin D supplementation (Fig. 4B: VitD^−/+^, *P* = 0.004). This observation was in the opposite direction to the effects of vitamin D deficiency (and subsequent supplementation) on BALF neutrophil and macrophage numbers (Fig. 1B).

### Vitamin D supplementation of initially vitamin D‐deficient mice increased expression of Vdr mRNA in the lung

Increased *Vdr* mRNAs were detected in the lungs of VitD^−/+^ mice, compared to mice fed the vitamin D‐containing diet (but not vitamin D‐deficient diet, *P* = 0.28) throughout life (Fig. 4C, *P* = 0.04).

### Vitamin D deficiency inhibited the activation of B cells in airway‐draining lymph nodes

**Figure 5:**
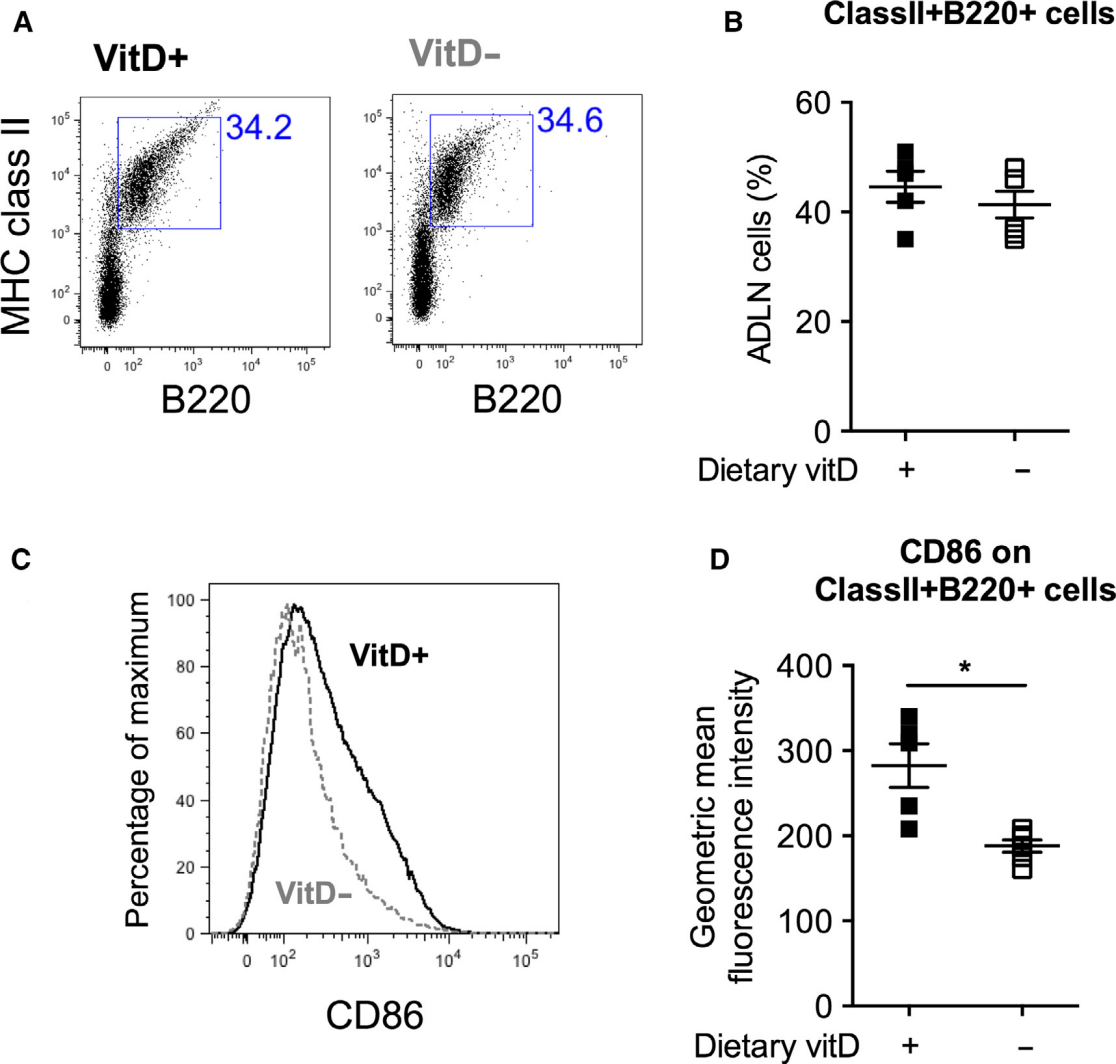
Vitamin D deficiency reduced the activation status of B cells in the airway‐draining lymph nodes. Female BALB/c mice (dams) were fed vitamin D‐supplemented (VitD^+^) or vitamin D‐nonsupplemented (VitD^−^) diets from 3 weeks of age and used to produce adult offspring. In (A), the proportion of B cells was determined using flow cytometry to select for MHC class II+ and B220+ cells, with the proportion of B cells (MHC class II+B220+) for each mouse shown in (B). In (C), CD86 expression is depicted for representative samples, with the level of expression of CD86 on MHC class II+B220+ cells shown in (D). Data are for 5–6 mice per treatment (mean ± SD, *P < 0.05, two‐way *t* test).

In vitro stimulation of B cells enriched from human blood with active 1,25(OH)_2_D has previously been shown to reduce the proportion of IgG‐secreting B cells, decreasing levels of IgG in tissue culture supernatants (Rolf et al. 2016). However, we observed an opposite effect with increased IgG levels in the BALF of VitD^+^ mice compared to VitD^−^ mice (Fig. 4B). Signaling through CD86 increases the capacity of B cells in regional airway‐draining lymph nodes (mediastinal) to produce IgG locally (and systemically) (Rau et al. 2009). We observed no difference in the proportion of B cells (B220+MHC class II+) in the airway‐draining lymph nodes of VitD^+^ mice compared to VitD^−^ mice (Fig. 5A, B). However, reduced expression of the activation marker CD86 was detected on B cells from VitD^−^ mice (Fig. 5C, D; *P* = 0.004, 33% reduction), which may explain the reduced IgG levels observed in the BALF of mice fed a vitamin D‐deficient diet.

## Discussion

In this study, we examined the effects of lifelong vitamin D deficiency and subsequent dietary supplementation on the integrity of the lung epithelium in naïve male BALB/c mice. There were no significant effects of vitamin D deficiency on most measures of lung epithelial integrity, including wet‐to‐dry lung weight, and the expression levels of tight junction proteins. Claudin‐1 protein levels were reduced in the lung epithelium of initially vitamin D‐deficient mice later supplemented with vitamin D (VitD^−/+^) compared to the other treatments; however, there was no other evidence that supported an effect on lung epithelial integrity in mice of this treatment. Supplementation with vitamin D also enhanced the expression of *Vdr* mRNA in the lung, which (as discussed below) may be linked with the capacity of dietary vitamin D to reduce lung inflammation as while vitamin D deficiency increased macrophage and neutrophil numbers in BALF, subsequent supplementation with vitamin D reversed this effect. An added benefit of maintaining sufficient vitamin D could be to boost B‐cell responses, as we also observed significantly increased IgG levels in the BALF of vitamin D‐replete mice, which was associated with increased expression of CD86 on B cells in the ADLN, and reduced lung inflammation (fewer macrophages and neutrophils in BALF).

The claudin family is composed of more than 23 proteins that interact with each other, and other proteins in a complex fashion to form the tight junction (Van Itallie and Anderson 2013). We observed reduced expression of claudin‐1 protein in airway epithelial cells of initially vitamin D‐deficient mice subsequently supplemented with vitamin D. Elevated claudin‐1 protein levels may be associated with poorer lung health; increased claudin‐1 protein was observed in airway smooth muscle cells from asthmatic patients, and in mice with OVA‐induced allergic airway disease, with claudin‐1 expression linked with increased airway remodeling (Fujita et al. 2011). Others have also shown that vitamin D can inhibit claudin expression in epithelial cells. For example, claudin‐2 protein was inhibited by in vitro treatment of duodenal cells with 1,25(OH)_2_D (Du et al. 2015). Other claudins can be negatively regulated by 1,25(OH)_2_D, including claudin‐5 (Won et al. 2015) and claudin‐16 (Kladnitsky et al. 2015); however, not all studies show this negative regulation (reviewed by Zhang et al. 2013), suggesting that the effects of 1,25(OH)_2_D might be context or site dependent and reflect the complex nature of the tight junction. Future studies may also choose to validate the reduced claudin‐1 protein levels we observed (using immunofluorescence) by other means (e.g., western blot), and measure the effects of vitamin D supplementation on the expression of other claudins and proteins associated with the tight junction.

The observed increased expression of *Vdr* mRNA in lungs of initially vitamin D‐deficient mice, then supplemented with vitamin D_3_ could be linked with the reduced expression of claudin‐1 in the lung epithelia of the same mice. In human intestinal epithelial cells, the promoter of the tight junction gene, *claudin‐2*, is transcriptionally activated by the VDR, potentially through a vitamin D response element that is regulated by 1,25(OH)_2_D (Zhang et al. 2015). Local levels of 1,25(OH)_2_D may increase in the lungs (improving its bioavailability) in response to supplementation of deficient mice with vitamin D_3_, increasing the expression of the VDR. Other studies have shown that mice with vitamin D deficiency had reduced VDR expression in the lungs (Agrawal et al. 2012). A caveat of our observations is that we did not examine VDR protein levels.

We did not observe an effect of vitamin D deficiency or subsequent supplementation with vitamin D_3_ on occludin expression by lung epithelial cells. Others have identified positive associations between serum 25(OH)D and occludin protein expression in the colonic mucosa (Meckel et al. 2016). Similarly, VDR^+/+^ mice had increased levels of the occludin protein in their corneas (Elizondo et al. 2014) and LPS‐injured lungs (Shi et al. 2016) compared to VDR^−/−^ mice. Interestingly, there was no difference in occludin protein or mRNA levels in uninjured lungs from VDR^+/+^ or VDR^−/−^ mice (Shi et al. 2016), suggesting that the VDR is protective during inflammation. Further treatment of VDR^−/−^ mice with the 1,25(OH)_2_D analog paricalcitol increased occludin levels, improved lung integrity, and reduced lung injury (Shi et al. 2016). These observations point to a vitamin D‐dependent pathway, whereby occludin maintains the lung epithelial barrier to prevent inflammation‐induced lung injury.

While we have focused on the effects of vitamin D on epithelium integrity and inflammation in the lungs, far more is currently known regarding the effects of vitamin D and the VDR on epithelial integrity in the gut. Vitamin D‐deficient (and hypocalcemic) C57Bl/6 female mice had increased epithelial barrier permeability and colonic hyperplasia compared to vitamin D‐replete mice both before and after infection with *Citrobacter rodentium* (Assa et al. 2014). Better colonic function was also associated with improved epithelial integrity in mice treated with 1,25(OH)_2_D, with colitis induced by dextran sulfate sodium (DSS). In these mice, intragastric 1,25(OH)_2_D prevented bacterial translocation to regional lymph nodes, improved epithelial permeability, and reduced intestinal crypt distortion induced by DSS (Kong et al. 2008; Zhao et al. 2012). Similarly, mice with intact expression of the VDR had less severe colitis and increased tight junction protein expression in the colon compared to VDR^−/−^ mice with DSS‐induced colitis (Kong et al. 2008). Epithelial‐specific overexpression of the human VDR increased expression of tight junction mRNAs in mice with colitis induced by a proinflammatory hapten (Liu et al. 2013). Reduced E‐cadherin was also observed in gut epithelial cells of CYP27B1^−/−^ mice compared to wild‐type mice (Ooi et al. 2013). CYP27B1^−/−^ mice do not express the 1*α*‐hydroxlase that makes active 1,25(OH)_2_D. These mice also had increased intestinal epithelial permeability when administered DSS, compared to wild‐type animals (Ooi et al. 2013). Higher maternal vitamin D levels reduced intestinal permeability in C57Bl/6J male offspring (Villa et al. 2016). In humans, vitamin D supplementation (2,000 IU/day, 3 months) of individuals with Crohn's disease prevented the deterioration of bowel permeability as compared to placebo‐treated subjects (Raftery et al. 2015). These findings highlight how various elements of the vitamin D pathway (including the VDR) are required for optimal epithelial integrity in the gut.

The mechanisms by which vitamin D improves gut epithelial health are linked with reduced inflammation. Indeed, dietary vitamin D (Assa et al. 2014), intact or increased expression of the VDR (Kong et al. 2008; Liu et al. 2013; Ooi et al. 2013), or treatment with active 1,25(OH)_2_D (Zhao et al. 2012) reduced the extent of gut inflammation observed in a number of the models described above. Other findings suggest that the capacity of vitamin D to regulate epithelial function and inflammation may be intertwined as pathways central to inflammation, which are activated by proinflammatory mediators, also have important roles in maintaining epithelial integrity (Du et al. 2015). Both 1,25(OH)_2_D and the VDR are required to suppress NF‐*κ*B‐related signaling pathways to maintain tight junctions (Du et al. 2015), and inhibit epithelial cell apoptosis (Liu et al. 2013) in the colon. However, in the current studies, it seemed that the emergence of inflammatory cells (e.g., macrophages and neutrophils) in the lungs of vitamin D‐deficient mice precluded any defects in epithelial integrity that might be induced by a challenge event (e.g., LPS or allergic airway disease).

Other studies suggest that vitamin D and the VDR may promote the capacity of tolerogenic dendritic cells (Ooi et al. 2013) and regulatory T cells (Gorman et al. 2007) to suppress inflammation and modify the microbiome (Assa et al. 2014; Wang et al. 2016) of the gut. Epidermal (skin) application of 1,25(OH)_2_D also enhances the suppressive capacity (Gorman et al. 2007, 2010) and proliferative activity (Ghoreishi et al. 2009) of Foxp3+ regulatory T cells, suppressing skin inflammation (Kivelevitch et al. 2013) and improving epidermal integrity compromised by tape stripping (Hong et al. 2010). Unexpectedly, we have recently observed reduced proportions of Foxp3+ regulatory T cells in the ADLN of vitamin D‐replete mice, compared to vitamin D‐deficient mice, with no differences observed in the lungs (Gorman et al. 2016), suggesting that dietary vitamin D may not induce immune tolerance in the lungs of naïve mice. In addition, we identified that serum levels of 25(OH)D inversely correlated with total bacterial and *Pseudomonas* OTUs in the lungs of naïve BALB/c mice (Roggenbuck et al. 2016). These observations appear to be independent of any major changes to the integrity of the lung epithelium.

In addition to promoting immune tolerance through Foxp3+ regulatory T cells and tolerogenic DCs, other immune pathways are modulated by vitamin D (Hart et al. 2011; Muehleisen and Gallo 2013). B cells are targets of vitamin D, and increase their expression of the VDR and CYP27B1 upon activation (reviewed by Rolf et al. 2016). We observed reduced levels of IgG in the BALF of vitamin D‐deficient mice, compared to mice fed a diet supplemented with vitamin D. In addition, reduced CD86 expression was observed on B cells in the ADLN of vitamin D‐deficient mice, compared to mice fed a vitamin D‐containing diet. However, these results do not corroborate other in vitro studies, which show that 1,25(OH)_2_D downregulates CD86 on B cells during activation (reviewed in Rolf et al. 2016). Increased presence of IgG in the lungs with dietary vitamin D may have benefits to prevent the colonization of pathogens (through processes such as IgG‐mediated opsonization). Immunoglobulin isotypes in the BALF were affected in different ways by vitamin D deficiency or vitamin D supplementation (of deficient mice). BALF levels of IgG and IgM levels were reduced in vitamin D‐deficient mice compared to vitamin D‐replete mice. IgM levels in BALF and serum were reduced in mice of the VitD^−/+^ treatment compared to VitD^+^ mice, suggesting that systemically IgM levels were reduced in VitD^−/+^ mice and that the reduced levels in BALF were not due to impaired alveolar leakage. However, further work is needed to clarify these relationships, as there was no significant positive correlation between serum and BALF IgM levels.

In conclusion, we report no beneficial effects on lung epithelial integrity by vitamin D supplementation of initially vitamin D‐deficient mice. However, signs of lung inflammation induced by deficiency were diminished by subsequent supplementation with vitamin D. Our findings highlight the importance of ongoing vitamin D supplementation trials that aim to reduce the severity of asthma, and other chronic lung diseases. Recent meta‐analyses suggest that vitamin D supplementation may reduce the likelihood of asthma exacerbation (Riverin et al. 2015; Martineau et al. 2016), and it will be important to determine whether treatment is more effective in individuals that are vitamin D deficient prior to commencing vitamin D supplementation.

## Conflict of Interest

None declared.
